# Supplementary material for: The Gelatinase Inhibitor ACT-03 Reduces Gliosis in the Rapid Kindling Rat Model of Epilepsy, and Attenuates Inflammation and Loss of Barrier Integrity In Vitro
Source: Biomedicines. 2022 Aug 29;10(9):2117. doi: 10.3390/biomedicines10092117 (PMC9495904; doi:10.3390/biomedicines10092117)
Supplement: Supplementary file 1 [file biomedicines-10-02117-s001.zip › biomedicines-1770570-supplementary.pdf]

Supplementary Materials

Supplementary Figure

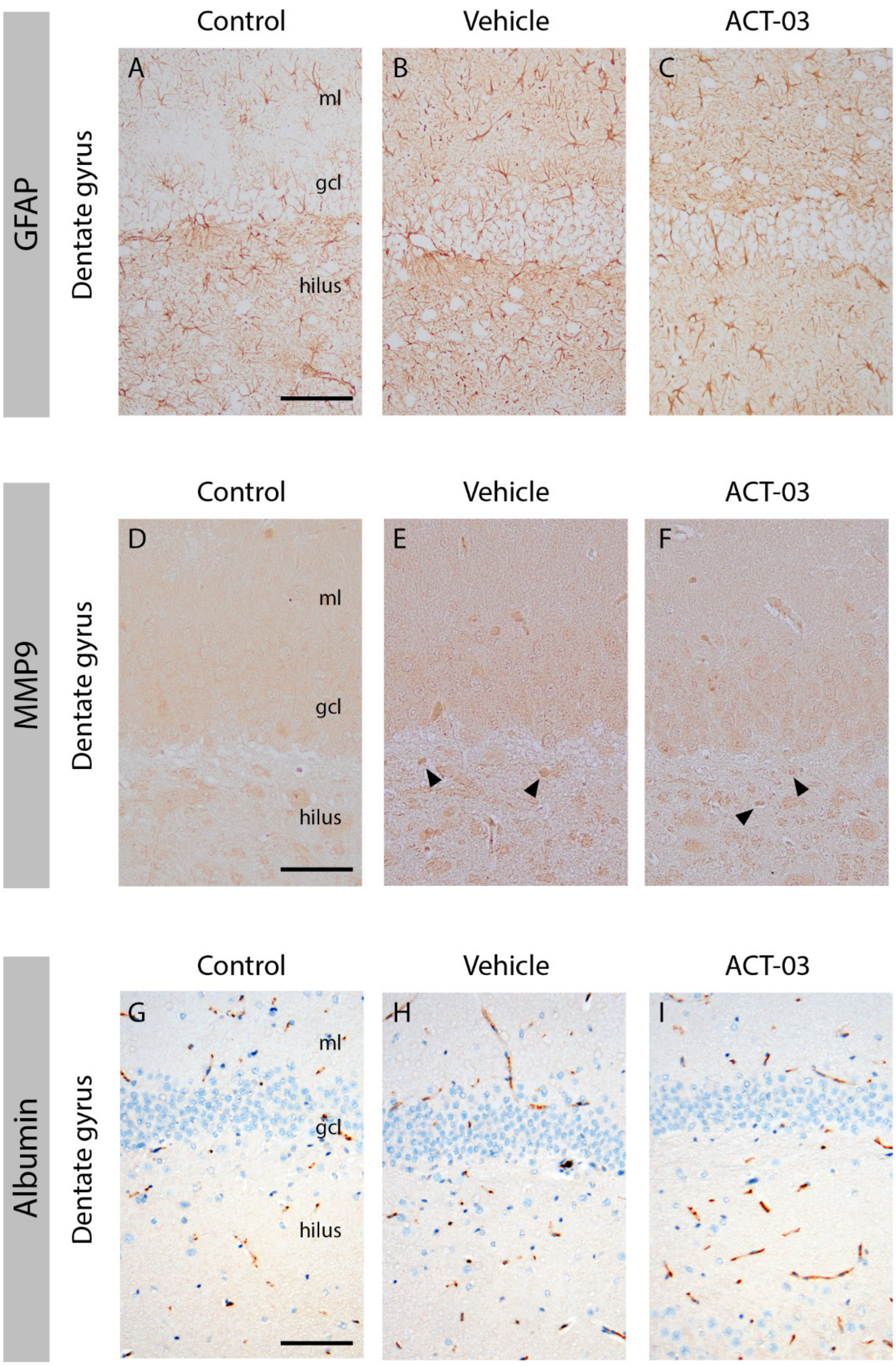

Supplementary Figure S1. Glial fibrillary acidic protein (GFAP), MMP9 and albumin staining was unchanged in ACT-03-treated kindled rats compared to vehicle-treated kindled rats. Representative images of GFAP immunohistochemistry (A-C) shows gliosis in vehicle-treated kindled animals and a tendency to less gliosis in the ventral hippocampus of ACT-03-treated kindled rats. MMP9 immunohistochemistry (D-F) shows constitutive

expression of MMP9 in neurons. Expression in glial cells (arrowheads) is also seen in kindled animals. Albumin immunohistochemistry (hematoxylin counterstained, G-I) of the dentate gyrus. Albumin positivity was seen inside blood vessels in all groups, extravascular albumin positivity was not observed. Error bar in A for A-C and D for D-F: 100µm; ml, molecular layer; gcl, granular cell layer.

Supplementary Tables

Supplementary Table S1. Semi-quantification of GFAP staining in rat hippocampus

	Ventral	Dorsal
**Control**	1	1
**Vehicle**	3 (2-3)	3
**ACT-03**	2 (2-3)	3

The number of GFAP-positive cells was scored on a 0-3 scale for the ventral and dorsal hippocampus (0 = no; 1 = sparse; 2 = moderate; 3 = high). Quantification score is given as median scores and variance within groups as minimum-maximum score (indicated in brackets).

Supplementary Table S2. Semi-quantification of MMP9 staining in rat hippocampus

**NEURONS**	*Ventral hippocampus*		*Dorsal hippocampus*	
	**DG**	**CA1**	**DG**	**CA1**
**Control**	6 (3-9)	6 (3-9)	6 (3-9)	6 (3-9)
**Vehicle**	6 (3-12)	6 (3-12)	6 (3-6)	6 (3-6)
**ACT-03**	6 (6-9)	6 (6-9)	6 (3-9)	6 (3-9)

**GLIA**	*Ventral hippocampus*		*Dorsal hippocampus*	
	**DG**	**CA1**	**DG**	**CA1**
**Control**	3 (2-8)	3 (0-8)	3 (0-8)	3 (0-8)
**Vehicle**	5 (3-8)*	3 (0-8)	6 (4-8)**	3 (0-4)
**ACT-03**	4 (3-8)*	3 (0-8)	4 (3-8)*	2.5 (0-4)

The immunoreactivity score (IRS) was calculated by multiplying the intensity score by the number of positive cell scores. The intensity of the staining was evaluated using a 1-4 scale (1: no; 2: weak; 3: moderate; 4: strong staining). The number of MMP9-positive cells was evaluated using a 0-3 scale (0 = no; 1 = sparse; 2 = moderate; 3 = high). The IRS is given as median scores and variance within groups as minimum-maximum score (indicated between brackets). Significantly higher than controls when indicated with *$p < 0.05$, **$p < 0.01$; Mann-Whitney U test.

Supplementary Methods

Animals

Adult male Sprague Dawley rats (ordered at 250 g, about 2 months of age, n=30, Harlan Netherlands, Horst, the Netherlands) were used for the rapid kindling model, as previously reported [1]. The rats were housed individually in a controlled environment (21±2°C; humidity 55±15%; lights on 08:00 AM - 8:00 PM; food and water available ad libitum) and acclimatized for one week after arrival. Animals were randomly assigned to different treatment groups; non-kindled controls (n=10), vehicle-treated kindled animals (n=10) and ACT-03-treated kindled animals (n=10).

Electrode implantation

Rats were anesthetized using a mixture of ketamine (74 mg/kg; Alfasan, Woerden, the Netherlands) and xylazine (11 mg/kg; Bayer AG, Leverkusen, Germany), injected intramuscularly. Implantation was performed using a stereotactic frame. Two pairs of insulated stainless-steel electrodes were implanted; into the left dentate gyrus for recording and in the angular bundle for stimulation. Electrodes were implanted under electrophysiological control as described previously [1]. Animals were housed individually after surgery for two weeks to recover. Subsequently, animals were transferred to their own recording cages (40x40x80 cm) and connected to a recording and stimulation system (NeuroData Digital Stimulator, Cygnus Technology Inc, USA). Animals were able to roam the cage freely using a shielded multi-strand cable and electrical swivel (Air Precision, Le Plessis Robinson, France).

EEG recording

The signals originating from the animals' headstages were fed through commutators and a filter (own design) and amplification unit (BR-20D Breakout Box, NPI electronic GmbH, Tamm, Germany). Hereafter, signals were sampled by a computer-controlled digitized card (NI USB-6255, National Instruments Netherlands, Woerden, the Netherlands) which operates in a synchronized way to control the stimulation patterns. Amplification of the EEG signals (10x) occurred within the headstage and signals were band-pass filtered (0.1-1000 Hz), and then digitized at 2 kHz (16 bit; 30.5 µV/bit) using in-house data acquisition software using MATLAB (MathWorks, Natick, MA, USA). EEG recording started 1 day before kindling stimulations to assess baseline EEG, and continued throughout the experiment (24/7). Stimulation was performed by the same NI USB-6255 multifunction I/O device. This device is able to deliver biphasic, bipolar voltage stimulation pulses (max 20 V) to the individual rats, multiplexed to several different rats by the use of two ERB-24 USB-based 24-channel electromechanical relay interface devices (Measurement Computing Corporation, Norton, MA, USA). Adequate artifact suppression in the EEG recordings was achieved by perfect synchronization between stimulation and recording. In-house data acquisition software using MATLAB (MathWorks, Natick, MA, USA) was programmed to both evoke field potentials as well as delivering the kindling stimulus. EEG signals were also analyzed using this software.

Kindling stimulation

Rats were placed in the EEG measurement cages for one week of habituation prior to stimulations. Applying field potentials to the rats, the proper kindling stimulation intensity was determined. Kindling stimulations were applied at 70% of the maximum intensity minus the minimum stimulus intensity needed to saturate the field potential. Hereafter, rats were kindled by delivering bipolar, biphasic 0.2 ms pulses (0.1 ms each), 50 Hz, for 10 sec, 12 times a day with 45-minute intervals, for three subsequent days (as shown in Suppl. Fig. 1A). The first kindling stimulation on each day was given 1 hour after drug treatment. Behavioral seizures were scored according to the Racine's scale [2]. On day 22, a re-test was performed, in absence of the drug, during which rats received 7 stimulations in total. Animals that did not have proper EEG signals or did not react with both behavioral-and EEG-seizure activity were excluded from the study (n=2 in vehicle group and n=2 in ACT-03-treated group). The following day, rats were sacrificed by decapitation following isoflurane-induced anesthesia.

Semi-quantification of GFAP and MMP9 immunohistochemistry

Analysis of GFAP was performed for the dorsal and ventral hippocampus of control and kindled rats. The number of GFAP positivity was scored on a 0-3 scale for both ventral and dorsal hippocampal

regions (in the dentate gyrus (DG) and CA1) (0 = no; 1 = sparse; 2 = moderate; 3 = high). A semi-quantitative analysis of MMP9 positive neurons and glia was performed on both ventral and dorsal hippocampal regions. The intensity of the staining was evaluated using a scale of 1-4 (1: no; 2: weak; 3: moderate; 4: strong staining). The score represents the predominant cell staining intensity found in each case. Additionally, the number of positive cells (0 = no; 1 = sparse; 2 = moderate; 3 = high) was also evaluated. The immunoreactivity score (IRS) was calculated by multiplying the intensity score by the number of positive cell score.

References

1. Broekaart, D. W.; Bertran, A.; Jia, S.; Korotkov, A.; Senkov, O.; Bongaarts, A.; Mills, J. D.; Anink, J. J.; Seco, J.; Baayen, J. C.; Idema, S.; Chabrol, E.; Becker, A. J.; Wadman, W. J.; Tarragó, T.; Gorter, J. A.; Aronica, E.; Prades, R.; Dityatev, A.; van Vliet, E. A., The matrix metalloproteinase inhibitor IPR-179 has antiseizure and antiepileptogenic effects. *The Journal of clinical investigation* **2021,** *131* (1).
2. Racine, R. J., Modification of seizure activity by electrical stimulation. II. Motor seizure. *Electroencephalogr Clin Neurophysiol* **1972,** *32* (3), 281-94.
